# Prince of Wales's Hospital Fund for London

**Published:** 1899-01-07

**Authors:** 


### THE INSTITUTIONAL WORKSHOP.

### PRINCE OF WALES'S HOSPITAL FUND FOR LONDON.

WITH the approval of His Royal Highness the President, the operations of the Fund have this year been limited to those hospitals lying within a radius of seven miles from Charing Cross which applied for a grant within the stated time, in answer to the advertisement inserted in the metropolitan journals. In the preliminary consideration of these applications the Visiting Committee received great help from the Central Hospital Council for London. Of those hospitals which applied and whose claims were considered, all but 12 receive grants. Of these 12 some were deemed by their existing income and by the grants already received from the Sunday and Saturday Funds to be this year adequately provided for. Others, though excellent institutions, did not appear to be hospitals in the strict sense; a few more were, by reason of their management, ruled to be undeserving of support. The details of the grants for this year are appended below:—

### GRANTS AND CONDITIONS.

To London Hospital, £5,000; the committee recommended that the grant be made on the same conditions as last year, and this applied to Guy's Hospital (which received £5,000), to St. Thomas's Hospital (which received £1,800), to Middlesex Hospital (which received £1,000), and to St. Mary's Hospital (which received £1,000). King's College Hospital, £1,000 and £475; the committee recommended that the annual grant of £1,000 be continued, and that a special grant of £475 be made towards re-flooring the wards. Westminster Hospital, £750; the committee recommended that the annual grant be continued on the same conditions as last year, and the same with regard to University College Hospital (receiving £1,400), Charing Cross Hospital (£1,000), Royal Free Hospital (£750), and Seamen's Hospital (£500). Hospital for Consumption, Brompton, £500; the committee recommended that this grant be made towards the cost of the proposed country branch and Convalescent Home, on condition that the balance of the estimated cost is provided and the work put in hand without any avoidable delay. Hospital for Sick Children, Great Ormond Street, W.C., £500; the committee recommended that this grant be continued on the same conditions as last year. City of London Hospital for Diseases of the Chest, Victoria Park, E., £1,000; the committee recommended that this grant be made in order to provide for the reopening of 15 of the 60 beds now closed. National Hospital for the Paralysed and Epileptic, £750; the committee recommended that this grant be continued on the same conditions as last year. Metropolitan Hospital, £500. Great Northern Central Hospital, £750; the committee recommended that this grant be made on condition that 15 of the 25 beds now closed are opened. German Hospital, £150. London Temperance Hospital, £500; the committee recommended that this grant be made on condition that the further sum necessary to open the closed ward (12 beds) is raised and the ward opened. West London Hospital, £500; the committee recommended that this grant be made in the expectation that the overcrowding in the old wards will be abolished, and that the suggestions of the visitors be communicated to the hospital authorities. French Hospital, £100. West Ham Hospital, £250. North-West London Hospital, £300. Mildmay Mission Hospital, £250. Bolingbroke Hospital, £250; the committee recommended that this grant be made towards the cost of a new accident ward on condition that the balance of the estimated cost is provided and the work put in hand without any avoidable delay. Miller Hospital, £200. Memorial Cottage Hospital, £50. Establishment for Gentlewomen, Harley Street, £75; the committee recommended that this grant be made only on the understanding that a properly constituted committee is elected forthwith. St. Saviour's Hospital, Osnaburgh Street, £50. Royal Hospital for Diseases of the Chest, City Road, £100. North London Hospital for Consumption, £1,000; the committee recommended that this grant be made on condition that 15 out of the 20 beds now closed are opened. East London Hospital for Children, £250; the committee recommended that this grant be made towards a new operating theatre. Alexandra Hospital for Children, £500. North-Eastern Hospital for Children, £250; the committee recommended that this grant be made in anticipation of improvements being made in the directions indicated by the visitors. Paddington Green Hospital for Children, £150. Royal Hospital for Children and Women, £100. Samaritan Free Hospital, £500; the committee recommended that attention be called, in making the grant, to the overcrowded state of the out-patient department, and that the suggestion be made that it might temporarily be remedied by the children as out-patients being referred to children's hospitals. Grosvenor Hospital for Women and Children, £100; the committee recommended that attention be called, in making this grant, to the high cost per in-patient per week. Hospital for Women, Soho Square, £350. Chelsea Hospital for Women, £300. Queen Charlotte's Hospital, £300. British Lying-in Hospital, £50; Royal London Ophthalmic Hospital, £450; the committee recommended that attention be called, in making this grant, to the substance of the visitors' recommendations. London Lock Hospital (Female), £500; the committee recommended that this grant be made on the understanding that the amount necessary for the reconstruction of the drainage of the hospital be provided and the work put in hand without any avoidable delay, and that the overcrowding in wards Nos. 1, 5, 7, 8, 9, and 10 reported by the visitors be abolished. St. Peter's Hospital for Stone, £50. London Homœopathic Hospital, £200. Total, £23,200—£8,300.

The grants recommended by the Convalescent Hospitals Committee were:

Metropolitan Convalescent Institution, 32, Sackville Street, Piccadilly, W., £200; Morley House Seaside Convalescent Home, St. Margaret's Bay, Dover, £200; Mary Wardell Convalescent Home, Stanmore, Middlesex, £100; Royal Sea Bathing Hospital, Margate, £100; Charing Cross Hospital, Convalescent Home at Limpsfield, £50; Chelsea Hospital for Women, Convalescent Home at St. Leonard's-on-Sea, £50; East London Hospital for Children, Convalescent Home being erected at Bognor, £50; King's College Hospital, Convalescent Home, Hemel Hempstead, £50; Middlesex Hospital, Convalescent Home at Clacton-on-Sea, £50; National Hospital for Paralysed and Epileptic, Convalescent Home at East Finchley, £50; Queen Charlotte's Hospital, Convalescent Home at Kilburn, £50; Metropolitan Hospital, Convalescent Home at Cranbrook, £25; Paddington Green Children's Hospital, Convalescent Home at Harrow, £25. Total, £1,000.

SUMMARY.

| | |
|---|---|
| Grants to Hospitals ... ... ... ... | £31,500 |
| Grants to Convalescent Hospitals ... ... | 1,000 |
| Grand Total ... ... ... ... | £32,500 |

Thus the £32,500 distributed this year is shared by 59 hospitals and convalescent homes. The result is that, in addition to supplying other specified needs, 57 more closed beds will be reopened, making, with 185 reopened last year, in all 242 closed beds not only reopened, but maintained through the Fund.

The 100 dozen pints of champagne presented by Messrs. Charles Heidsieck have been stored by Messrs. Thomas Trapp and Sons, who have undertaken to deliver them to the various hospitals free of cost. This wine is divided among the hospitals receiving grants, approximately in proportion to the number of beds occupied, exclusive of pay beds.

